# Genomics insights into production of 2-methylisoborneol and a putative cyanobactin by *Planktothricoides* sp. SR001

**DOI:** 10.1186/s40793-017-0247-1

**Published:** 2017-06-05

**Authors:** Shu Harn Te, Boon Fei Tan, Chek Yin Boo, Janelle Renee Thompson, Karina Yew-Hoong Gin

**Affiliations:** 1 NUS Environmental Research Institute, National University of Singapore, Singapore, Singapore; 2 Singapore Centre for Environmental Sensing and Modelling, Singapore-MIT Alliance for Research and Technology Centre, Singapore, Singapore; 3 Department of Civil and Environmental Engineering, Massachusetts Institute of Technology, Cambridge, MA USA; 4 Department of Civil and Environmental Engineering, National University of Singapore, Singapore, Singapore

**Keywords:** Phormidiaceae, *Planktothricoides*, 2-methylisoborneol, Viridisamide A, Cyanobactin

## Abstract

*Planktothricoides* is a free-living filamentous cyanobacterium belonging to the order Oscillatoriales and the family Phormidiaceae, capable of forming bloom in fresh and brackish waters. A unicyanobacterial non-axenic culture dominated by *Planktothricoides* sp. SR001 was obtained from a freshwater reservoir in Singapore. The draft genome presented here is the first tropical freshwater *Planktothricoides* sp. ever sequenced. The genome of 7.0Mbp contains 5,776 genes predicted using the JGI IMG pipeline. The whole genome sequence allows identification of genes encoding for nitrogen-fixation, accessory photosynthetic pigments and biosynthesis of an off-flavor compound, 2-methylisoborneol, which has been experimentally verified here based on metabolite detection. In addition, strain SR001 genome contains an operon putatively involved in the production of a linear tripeptide cyanobactin related to viridisamide A and aeruginosamide, with the later known to possess anti-microbial or cytotoxic effect.

## Introduction

Managing cyanobacterial blooms is a growing concern worldwide due to increasing anthropogenic pollution and climate change that lead to eutrophication of marine, estuarine and fresh waters [1, 2]. Secondary metabolites produced by cyanobacteria are one of the emerging pollutants causing environmental degradation, economic losses and negative impacts on drinking and recreational waters [3–5]. Amongst the metabolites, odiferous terpenes are commonly detected in many cyanobacterial species, for example geosmin and 2-MIB which give earthy and muddy smells, are responsible for most of the taste and odor issues for water resources and subverting consumers’ confidence on the safety of treated water [6, 7]. Cyanobacterial toxins such as microcystins, cylindrospermopsins and saxitoxins, produced via the nonribosomal peptide synthetase or polyketide synthase, have been shown to cause intoxication cases in livestock and human [8]. Other than non-ribosomal peptides and polyketides, some cyanobacteria also produce bioactive compounds such as cyanobactins via post-ribosomal peptide synthesis.

Many cyanobacterial genera, such as *Aphanizomenon*, *Oscillatoria*, *Phormidium*, *Lyngbya* *,* *Pseudanabaena*, *Planktothrix* and *Planktothricoides* identified as the common off-flavor producers [6, 9], are also commonly found in freshwater bodies in Singapore. *Planktothricoides* is a bloom-forming planktonic-filamentous cyanobacterium which occurs naturally in freshwater and estuarine aquatic systems [10]. The genus was originally classified as *Planktothrix* under the family of Phormidiaceae due to their high morphological similarity; but it was later designated as a new genus because they are phylogenetically distinct cyanobacteria based on the 16S rRNA gene analysis [11]. *Planktothricoides* spp. have been occasionally detected in cyanobacterial blooms, either as dominating or co-occurring taxa [12–14]. They can produce taste-and-odor compounds (e.g. 2-MIB) and substances toxic to aquatic biota [15]. We report here the first draft genome of *Planktothricoides* sp. (SR001) which was isolated from a Singapore freshwater reservoir to facilitate molecular and physiological characterizations for a better understanding of their ecological roles in aquatic ecosystems for future study.

## Organism Information

### Classification and features

*Planktothricoides* sp. SR001 examined in this study was isolated from a reservoir located at the north-east part of Singapore. The reservoir receives water from its catchment with a mixed land use comprised of residential, industry and reserved lands. The water body was under eutrophic or hypereutrophic state (Carlson trophic index 63-75) characterized with high levels of chl-a and total phosphorus [16]. Two off-flavor compounds, 2-MIB and geosmin, exhibited concentration range from undetectable to 53.1 ng/L in reservoir water, frequently exceeded the olfactory thresholds of 4 – 10 ng/L for drinking water [17]. The phytoplankton community was dominated by cyanobacteria including genera capable of odor synthesis such as *Pseudanabaena*, *Planktothrix* and *Planktothricoides* [18, 19]. Isolation attempts were carried out to capture species responsible for off-flavor production. To obtain unicyanobacterial culture, grab water samples collected from the reservoir were examined under an inverted microscope (Leica DFC450 C) to identify target cyanobacteria. Filaments of *Planktothricoides* were picked using a sterile pipette and washed with sterile water before transferring into nutrient-enriched MLA medium [20]. After multiple transfers, a unicyanobacterial culture containing *Planktothricoides* as the dominant species was obtained for morphological and genomic characterization.

**Fig. 1:**
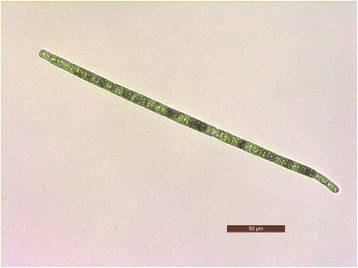
Photomicrograph of a trichome of *Planktothricoides* sp. SR001 under bright field and 40× magnification. Cell attenuation was observed at the end of trichome

**Fig. 2:**
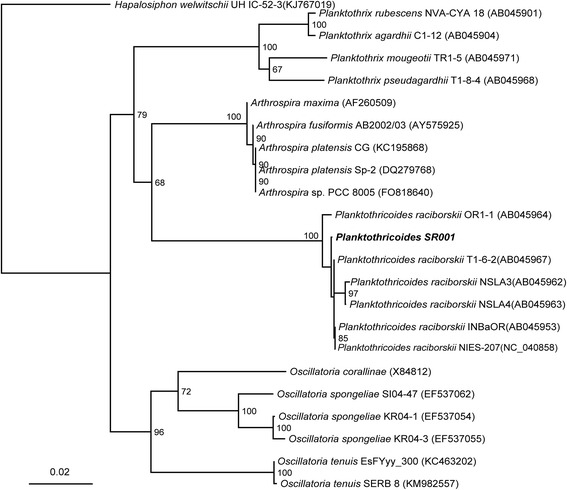
Neighbor joining tree of the 16S rRNA gene of strain SR001 and selected 16S rRNA sequences of the *Oscillatoriales*. All 16S rRNA sequences were aligned using MUSCLE [45], manually curated, following which neighbor joining tree was constructed using the Tamura-Nei Model. Bootstrap values are labelled in each branch node and the 16S rRNA of *Hapalosiphon welwitschii* UH IC-52-3 was used as the outgroup

Morphological identification of *Planktothricoides* sp. SR001 was determined based on the common morphology characteristics for Phormidiaceae family (Table 1), i.e. filaments are solitary, straight, free-floating and unbranched; cells in the filament have cylindrical shape; are shorter than wide and similar in shape [21]. This was followed by intergeneric identification based on phenotypic features of which the end of the trichome (filament of plankton) is attenuated and without calyptra (Fig. 1), differentiating *Planktothricoides* from the genus *Planktothrix* [10]. The average filament length was 282.4 (±93.9) μm; width of individual cell was 8.13 (±0.92) μm and length was 5.48 (±2.01) μm; and cell width to length ratio was 1.68 (± 0.65) μm. Gas vesicles were spread along the filament near the edge of the cell. The single copy 16S rRNA gene of 1497 bp (locus tag:AM228_RS28415) identified for strain SR001 is >99% identical to those in different strains of *Planktothricoides raciborskii* (*e.g.,* strain NIES-207, NR_040858.1), and form a congruent monophyletic clade with other *Planktothricoides* strains but is distinctive from clades containing *Planktothrix* spp., *Arthrospira* spp. and *Oscillatoria* spp. (Fig. 2).

**Table 1:** Classification and general features of *Planktothricoides* strain SR001 [38]

MIGS ID	Property	Term	Evidence code^a^
	Classification	Domain bacteria	TAS [39]
		Phylum *Cyanobacteria*	TAS [40]
		Class *Cyanophyceae*	TAS [41]
		Order *Oscillatoriales*	TAS [42]
		Family*Phormidiaceae*	TAS [21]
		Genus *Planktothricoides*	TAS [11]
		Species Unknown	
		Strain SR001	
	Gram stain	Negative	TAS [43]
	Cell shape	Filamentous / thallous	IDA
	Motility	Motile / free-floating	IDA
	Sporulation	Not reported	
	Temperature range	Not reported	
	Optimum temperature	Not reported	
	pH range; Optimum	Not reported	
	Carbon source	Phototrophic	IDA
MIGS-6	Habitat	Freshwater	IDA
MIGS-6.3	Salinity	0.015% NaCl (w/v)	
MIGS-22	Oxygen requirement	Aerobic	IDA
MIGS-15	Biotic relationship	Free-living	IDA
MIGS-14	Pathogenicity	Non-pathogen	IDA
MIGS-4	Geographic location	North-eastern region, Singapore	IDA
MIGS-5	Sample collection	March 2014	IDA
MIGS-4.1	Latitude	1.401577	IDA
MIGS-4.2	Longitude	103.927010	IDA
MIGS-4.4	Altitude	Not Applicable	

^a^Evidence codes - IDA: Inferred from Direct Assay; TAS: Traceable Author Statement (i.e., a direct report exists in the literature); NAS: Non-traceable Author Statement (i.e., not directly observed for the living, isolated sample, but based on a generally accepted property for the species, or anecdotal evidence). These evidence codes are from the Gene Ontology project [44]

#### Extended feature descriptions

**Fig. 3:**
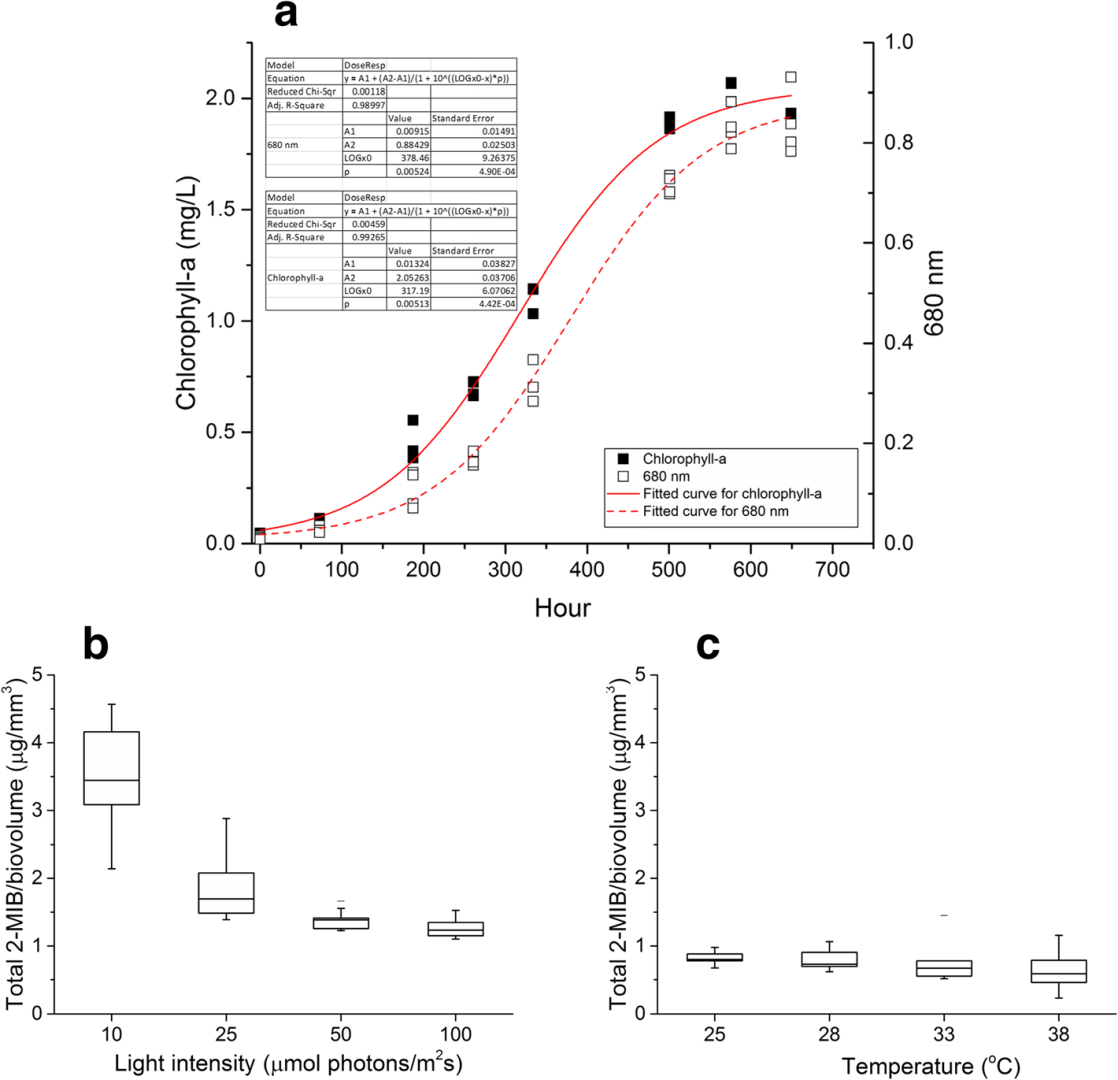
**a** Growth curves of *Planktothricoides* sp. SR001 fitted with dose-response model. Estimated growth rates are 0.00524 h^-1^ and 0.00513 h^-1^ based on optical density and chl-a, respectively; **b** Changes of 2-MIB content for *Planktothricoides* sp. SR001 under different light intensity and, **c** temperature conditions. 2-MIB contents are normalized to cell biovolume

Strain SR001 was sub-cultured through multiple transfers in MLA medium containing 6.2 mg phosphorus and 28 mg nitrogen per liter of medium. Incubation was conducted in a plant growth chamber (Percival) at 25 °C with light intensity 24.5 ± 2.0 μmol photons m^-2^s^-1^ and a dark/light cycle of 12 hours. The growth rate of strain SR001 was monitored spectrophotometrically with optical density at 680 nm, and also with biomass inferred from chl-a concentration. Both measurements demonstrated similar growth rates of 0.12 day^-1^ as illustrated in Fig. 3. The presence of accessory photosynthetic pigments commonly found in cyanobacteria including PC, APC and PE were assessed using methods described previously [22]. All three phycobilin pigments including PE, which are not found in earlier study of *Planktothricoides* [11], were detected in the late-exponential-phase culture with a PC:APC:PE:chl-a ratio of 1.4:2.8:0.7:1.0. Biochemical analysis of two cyanobacterial toxins, microcystins and cylindrospermopsin, were tested negative for strain SR001 using commercial ELISA kits (Abraxis, LLC). However, metabolite profiling using a GC-MS/MS triple quadrupole system (Agilent 7000 GC QQQ) with automated SPME extraction [7] detected 2-MIB but not geosmin during culture growth. Laboratory experiments were conducted to investigate the effects of environmental variables on strain SR001, as studies have shown that light intensity and temperature could alter the off-flavor production rates of cyanobacteria [23, 24]. Triplicate cultures cultivated under different light and temperature conditions were sampled three times during exponential phase, and 2-MIB concentration and cell biovolume were measured. It is worth noting that the culture of strain SR001 was able to tolerate a wide range of light intensity and temperature differences – from 10 to 100 μmol photons m^-2^ s^-1^ and from 18 to 38 °C. Significant reduction in 2-MIB was observed when light intensity increased from 10 to 50 μmol photons m^-2^ s^-1^ as illustrated in Fig. 3B. A 2-fold decrease in 2-MIB per biovolume was detected when light intensity doubled (independent T-test, *P* < 0.05) but no further decrease was found thereafter. In contrast, no significant difference (independent T-test, *P* > 0.05) in 2-MIB production was observed for the temperature range of 25 – 38 °C (Fig. 3c), indicating that the effect of temperature on the 2-MIB content per biovolume of strain SR001 was negligible.

## Genome sequencing information

### Genome project history

The project information and the associated MIGS 2.0 compliance [25] are provided in Table 2. Strain SR001 was selected for sequencing because it is capable of producing 2-MIB, an off-flavor which is known to reduce water palatability. Furthermore, the genome of *Planktothricoides* is currently underrepresented in public database. This work provides a standard draft genome, of which the assembled contigs have been deposited in NCBI database under the accession LIUQ00000000.

**Table 2:** Project information

MIGS ID	Property	Term
MIGS 31	Finishing quality	Draft
MIGS-28	Libraries used	Illumina Truseq Nano DNA Library Prep Kit
MIGS 29	Sequencing platforms	HiSeq Rapid V2 sequencing Run
MIGS 31.2	Fold coverage	150
MIGS 30	Assemblers	CLC Genomics Workbench 8.0
MIGS 32	Gene calling method	Prodigal
	Locus Tag	AM228
	Genbank ID	LIUQ00000000
	GenBank Date of Release	August 31, 2015
	GOLD ID	Ga0099329
	BIOPROJECT	PRJNA29364
MIGS13	Project relevance	Cyanobacterial ecology, Environmental

### Growth conditions and genomic DNA preparation

Our laboratory observation demonstrated that *Planktothricoides* sp. SR001 was able to grow in nitrogen-free MLA media, consistent with the genus of *Oscillatoria* in the same Order (Oscillatoriales) [26]. However, this might not be a generic physiological feature for all *Planktothricoides* as studies have shown that nitrogen fixing capacity is strain-dependent for *Planktothrix*, another member of *Oscillatoriales* [27, 28]. Individual trichomes were grown in nitrogen-free MLA media to select against non-nitrogen-fixing species. Strong association of strain SR001 with co-occurring heterotrophic bacteria resulted in a non-axenic unicyanobacterial culture which was maintained in MLA media incubated at 25 °C with a light intensity of 20 ± 5 μmol photons m^-2^s^-1^. Total DNA was isolated from the culture fluid using MO BIO PowerWater DNA Isolation Kit (MO BIO), following which the DNA quality and concentration were determined using Qubit 3.0 (Invitrogen).

### Genome sequencing and assembly

The total isolated DNA was used in the construction of a paired-end library using a Illumina TruSeq Nano DNA Library Prep Kit with an insert size of 550 bp, and subsequently sequenced with Illumina HiSeq 2000 applying the 250 bp paired-end sequencing protocol at Singapore Centre for Environmental Life Sciences Engineering. Adaptors and reads with quality score <0.01 and length <150 bp were removed using CLC Genomics Workbench V.8 (CLC-Bio, USA), yielded 9,839,009 paired-reads with average read length of 251 bp. The reads were then subjected to *de novo* assembly with CLC Genomics Workbench V8.0 using default kmer size. The mini-metagenome was assembled into 5,572 scaffolds (764 - 1,110,006 bp) with mean lengths (N50) of 86,064 bp and average length of 8,294 bp. The genome of strain SR001 was extracted from this mini-metagenome using MetaBAT [29], after which the extracted genome was confirmed for completeness and purity using CheckM [30]; thus revealing that the genome has 100% coverage of single copy genes and no evidence for sequence contamination or intra-strain genomic heterogeneity.

### Genome annotation

Gene prediction was performed using Prodigal [31] as part of the Joint Genomic Institute IMG automated genome annotation pipeline [32] and the NCBI Prokaryotic Genome Annotation Pipeline [33]. Additionally, gene clusters encoding secondary metabolite biosynthesis were predicted using AntiSMASH 3.0 [34].

## Genome Properties

The draft genome of 43.5% GC is 7.0 Mbp contained in 165 scaffolds (1017 – 297,434 bp; Table 3). The N50 and L50 of the 165 scaffolds are 108,940 and 22, respectively. Annotation using the NCBI Prokaryotic Genome Annotation Pipeline [33] predicted 5,776 total genes (Table 3). Complete genome statistics and COG annotation of protein coding genes are presented in Tables 3 and 4, respectively.

**Table 3:** Genome statistics

Attribute	Value	% of total
Genome size (bp)	7,066,705	100.0
DNA coding (bp)	5,499,857	77.8
DNA G + C (bp)	3,066,802	43.4
DNA scaffolds	165	-
Total genes	5,776	100.0
Protein coding genes	5,049	87.4
RNA genes^a^	53	0.9
Pseudo genes^a^	676	11.7
Genes with function prediction^b^	3,816	64.1
Genes assigned to COGs^b^	2,714	45.6
Genes with Pfam domains^b^	4,079	68.6
Genes with signal peptides^b^	159	2.7
Genes with transmembrane helices^b^	1,204	20.2
CRISPR repeats^a^	12	-

^a^Genome statistics obtained using the NCBI Prokaryotic Genome Annotation Pipeline [33]

^b^Genome statistics obtained using the JGI IMG pipeline [32]

**Table 4:** Number of genes associated with general COG functional categories

Code	Value	%age	Description
J	189	6.33	Translation, ribosomal structure and biogenesis
A	1	0.03	RNA processing and modification
K	94	3.15	Transcription
L	52	1.71	Replication, recombination and repair
B	2	0.07	Chromatin structure and dynamics
D	32	1.07	Cell cycle control, Cell division, chromosome partitioning
V	120	4.02	Defense mechanisms
T	241	8.07	Signal transduction mechanisms
M	212	7.1	Cell wall/membrane biogenesis
N	40	1.34	Cell motility
U	32	1.07	Intracellular trafficking and secretion
O	151	5.05	Posttranslational modification, protein turnover, chaperones
C	137	4.59	Energy production and conversion
G	116	3.88	Carbohydrate transport and metabolism
E	197	6.59	Amino acid transport and metabolism
F	72	2.41	Nucleotide transport and metabolism
H	185	6.19	Coenzyme transport and metabolism
I	75	2.51	Lipid transport and metabolism
P	157	5.25	Inorganic ion transport and metabolism
Q	58	1.94	Secondary metabolites biosynthesis, transport and catabolism
R	479	16.03	General function prediction only
S	222	7.43	Function unknown
-	3236	54.39	Not in COGs

The total is based on the total number of protein coding genes in the genome COG was obtained from the JGI IMG pipeline [32]

## Insights from the genome sequence

**Fig. 4:**
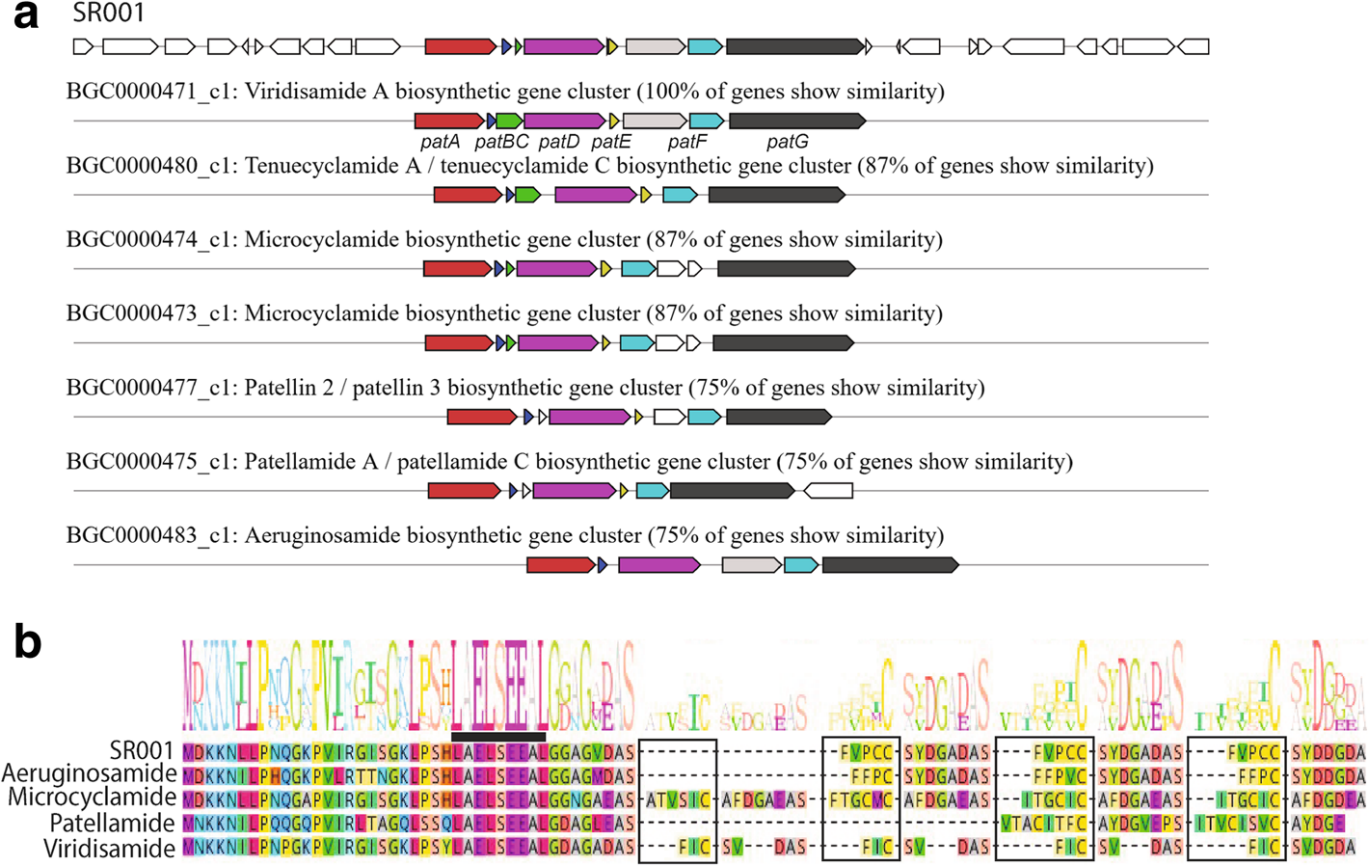
**a** Gene cluster in the genome of strain SR001 encoding biosynthesis of a putative cyanobactin related to viridisamide A. The *patABCDEFG* labelled for viridisamide A was named according to Fig. 4 a [35], and their predicted functions are listed in Table 5; **b** Alignment of cyanobactin precursor peptide according to [35]. Conserved motif LAELSEE is underlined, whereas conserved variable regions that are cleaved to form final cyanobactin are *boxed*

*Planktothricoides* is an important cyanobacterial species as several members of this genus are known to produce taste-and-odor compounds, as well as toxins that are harmful to aquatic biota [15]. Using antiSMASH 3.0.5 [34], complete gene clusters encoding biosynthesis of 2-MIB was detected in the genome of strain SR001 (Fig. 4A). In addition, a putative cyanobactin gene cluster with identical gene organization to the reference of viridisamide A was also found in the genome (Fig. 4a). No other toxins/off-flavors – *i.e*. microcystin, geosmin and cylindrospermopsin genes were detected using antiSMASH or tBLASTn using reference genes. The 2-MIB biosynthesis gene cluster contains homologous *cnbA*, *mtf, mic*, and *cnbB* genes [AM228_RS20060 to AM228_RS20075] with amino acid similarity of 85-93% compared to those detected in *Planktothricoides raciborskii* CHAB3331 (HQ830028). This finding is consistent with the detection of 2-MIB metabolite in strain SR001 culture fluids (Fig. 3b and c).

The putative viridisamide A gene cluster detected in strain SR001 contains eight genes with >60% amino acid similarity to those in the viridisamide A gene cluster first described for *Oscillatoria* *nigro-viridis* PCC 7112 [35]. Viridisamide A is a linear tripeptide (cyanobactin) and the organization of the gene cluster encoding for this cyanobactin is highly identical to that encoding for aeruginosamide identified in *Microcystis* PCC 9432 [35]. The leader sequences of the precursor peptides of viridisamide A, aeruginosamide and several other cyanobactins are highly conserved, although each sequence uniquely contains more than one variable core region that are modified and cleaved to form the final structural variants [35]. Evidently, the leader sequence of the precursor peptide in strain SR001 (AM228_RS10425) is also highly conserved compared to other cyanobactins and contains the highly-conserved motif LAELSEE in the leader sequence (Fig. 4b and Table 5). The core variable regions of the precursor peptide of strain SR001, however, are distinctive from those of viridisamide A and aeruginosamide (Fig. 4b); thus, suggesting that the cyanobactin produced is likely to be structurally different from the two linear cyanobactins. The final structures of this cyanobactin of strain SR001 is currently unknown. Like the gene clusters encoding viridisamide A and aeruginosamide [35], the putative cyanobactin gene cluster contains genes predicted to encode for thiazoline oxidase adjacent to a predicted c-terminal protease gene, thus suggesting that the cyanobactins of strain SR001 may contain a c-terminal bound to a thiazole. The functions of both viridisamide A and aeruginosamide have not been established.

**Table 5:** Gene cluster encoding cyanobactins detected in strain SR001 genome

Gene name^a^	Locus tag	Predicted function	Amino acid identity (%)	
			*Oscillatoria nigro-viridis* PCC 7112	*Microcystis aeruginosa* PCC9432
PatA	AM228_RS	N-terminal protease	90	73
PatB	AM228_RS	Hypothetical	82	92
PatC	AM228_RS	Hypothetical	78	44
PatD	AM228_RS	Heterocyclase	83	88
PatE	AM228_RS	Precursor	84	84
PatF1	AM228_RS	Methyltransferase	76	95
PatF2	AM228_RS	Putative prenyl transferase	73	83
PatG	AM228_RS	C-terminal protease/Thiazoline oxidase	80	75

^a^Naming according to [35]

Strain SR001 was isolated from a surface water sample and is likely a free-living planktonic cyanobacterial species. The genome carries multiple genes essential for movement within the water column including genes predicted to encode for gas vesicles important for buoyancy regulation [36], and pilus and twitching motility important for photo- and chemotaxis [37]. Energy is primarily derived through photosynthesis, with a predicted capability to harvest a broad spectrum of sunlight with different wavelengths, based on annotation of genes encoding alpha- and beta-subunits of phycocyanin (e.g., AM228_RS09220 and AM228_RS09225) and allo-phycocyanin (e.g., AM228_RS19895 and AM228_RS19900). The presence of different pigmentation likely confers ecological advantage for competitive growth in environments with fluctuating sunlight. Nitrogen is likely derived through N_2_ fixation, under some circumstances evidenced by annotation of multiple nitrogenase genes in the genome (*e.g.,* AM228_RS02340, AM228_RS22395), and growth in nitrogen-free media. Genes for utilization of additional nitrogen sources are predicted in the genome including ammonium [ammomium transporters, e.g., AM_SR11610], urea [urease (e.g., *ureABCDEF,* AM228_RS18855 to RS18880); urea transporter (RS18565 to RS1885650)] and nitrate (nitrate transporters, e.g., AM228_RS00860), indicating the strain is versatile in utilizing different nitrogen sources.

## Conclusions

This first draft genome sequence of *Planktothricoides* sp. will facilitate genetic insights into the genus of *Planktothricoides* which is currently under-described. The bioinformatic analysis revealed gene clusters encoding for nitrogenases, 2-MIB, PC, APC, which are in agreement with experimental data or physiological observations. In addition, a putative cyanobactin, likely related to viridisamide, was detected in the genome. Presence of genes encoding for nitrate, ammonia and urea transporters together with nitrogenases indicate that strain SR001 has evolved a variety of strategies that allow them to grow with different nitrogen sources. The genome presented here enables sequence analysis and comparative genomics to drive further research on the ecology and physiology of cyanobacterial strains that may impact water quality.

## Abbreviations

2-MIB: 2-methylisoborneol
APC: Allo-phycocyanin
chl-a: Chlorophyll-a
L50: Number of scaffolds whose summed length is N50
N50: Mean of contig/scaffold lengths
PC: Phycocyanin
PE: Phycoerythrin
